# Activation of Erk in ileal epithelial cells engaged in ischemic-injury repair

**DOI:** 10.1038/s41598-017-16714-6

**Published:** 2017-11-28

**Authors:** Haruna Takeda, Etsuko Kiyokawa

**Affiliations:** 1 Department of Oncologic Pathology, School of Medicine, Kanazawa Medical University, Ishikawa, 920-0293 Japan; 2 Present Address: Division of Genetics, Cancer Research Institute, Kanazawa University, Ishikawa, Japan

## Abstract

Intestinal epithelial cells function as a barrier to protect our body from various agents; therefore, any damage to these cells must be immediately repaired. Several *in vivo* and *vitro* studies have shown the involvement of Erk (extracellular signal-regulated kinase) in the regeneration process; however, the spatial regulation of Erk related to tissue morphology has not been well documented. Using two-photon microscopy and mice carrying a Förster resonance energy transfer-based biosensor, we here monitored the Erk activity in the ileal epithelial cells of living mice. Forty-eight h after ischemia-induced injury, epithelial cells were observed as a monolayer covering the injured area. The Erk activity in these cells was higher than that in the epithelial cells at the surrounding crypts, and treatment with an epidermal growth factor receptor inhibitor cancelled the higher Erk activity. The resealing epithelial cells were not in the G_2_/M phase of the cell cycle, and Yap (Yes-associated protein) was localized to the nucleus. Immunostaining of intestinal ulcers from patients revealed ERK phosphorylation and nucleus localization of YAP without Ki-67 staining in the resealing epithelial cells. These findings led us to propose that the YAP-EGFR-ERK axis is involved in migration, but not in proliferation, of the resealing epithelial cells.

## Introduction

The surface of the digestive tract is covered by a monolayer of epithelial cells that functions as a physical barrier to protect the body from pathogens and dietary substances^1^. Several pathological and iatrogenic conditions are known to cause injury to epithelial cells, such as chronic inflammation, ischemia, irradiation, and biopsy. After injury, the integrity of epithelial cells is re-established. To understand the cellular and molecular mechanisms that regulate regeneration of the injured area, various animal models have been established, including luminal acid exposure in the rabbit duodenum^2^ and colon^3^, endoscopic biopsy in the mouse colon^4,5^, addition of dextran sodium sulfate to the drinking water of mice and rats^6^, and irradiation of the mouse colon^7^. Since the speed and mode of epithelial repair are influenced by the cause, degree, and location of the injuries, we should be careful when combining the results from different models.

Ischemic damage is one of the major causes of intestinal ulcer or perforation^8^. The affected area in the intestine can range from a mucosal, to a mural, to a transmural infarction. The mucosal and mural infarctions often are secondary to hypo-perfusion, and associated with cardiac failure, shock, or dehydration. To induce ischemia or infarctions in animal experiments, complete or segmental vascular occlusion, artery ligation and embolization have been used^9^. Occlusion induces the initial hypoxic injury followed by the second, reperfusion phase. In the latter phase, blood restoration induces major damage with various types of cytokine production. Despite this severe situation, the epithelial cells reseal the injured area, and the failure in repair causes infection and inflammation, which can be fatal. It is therefore important to understand the mechanisms by which the epithelial cells migrate and cover the injured area. For this purpose, several *in vitro* systems using cultured cell lines from the intestine have been established^10^. However, the *in vitro* microenvironment has still not been fully reconstituted. In addition, the *in vivo* imaging of the intestine has not been achieved, due to the lack of techniques for *in vivo* experiments.

The recent development of two-photon excitation microscopy (TPM) enables us to observe the single cell dynamics deep within living animals^11^. In addition, biosensors based on Förster resonance energy transfer (FRET) have been developed for several kinases to monitor the activity in real time. For example, a FRET biosensor for extracellular-signal-regulated kinase (Erk), EKAR (extracellular signal-regulated kinase activity reporter), was generated^12^, followed by an improved version, EKAR-EV, with better sensitivity and dynamic range^2^. Finally, mice carrying EKAR-EV were generated to monitor the Erk activity *in vivo* ^13^. In the regeneration process of the wound in the skin, live-imaging by TPM showed that Erk activation propagates as a trigger wave in parallel to the wound edge^14^. Although it has been reported that an inhibitor treatment against Erk in mice increased apoptosis and decreased proliferation to worsen intestinal ischemia/reperfusion injury^15^, it has not been determined how Erk activity in the intestine is regulated upon injury. Focusing on the epithelial resealing process after ischemia-induced injury, we here observed that Erk is activated in the ileal epithelial cells covering the injured area.

## Results

### Establishment of a mouse model for Erk activities in the intestinal epithelial cells

**Figure 1:**
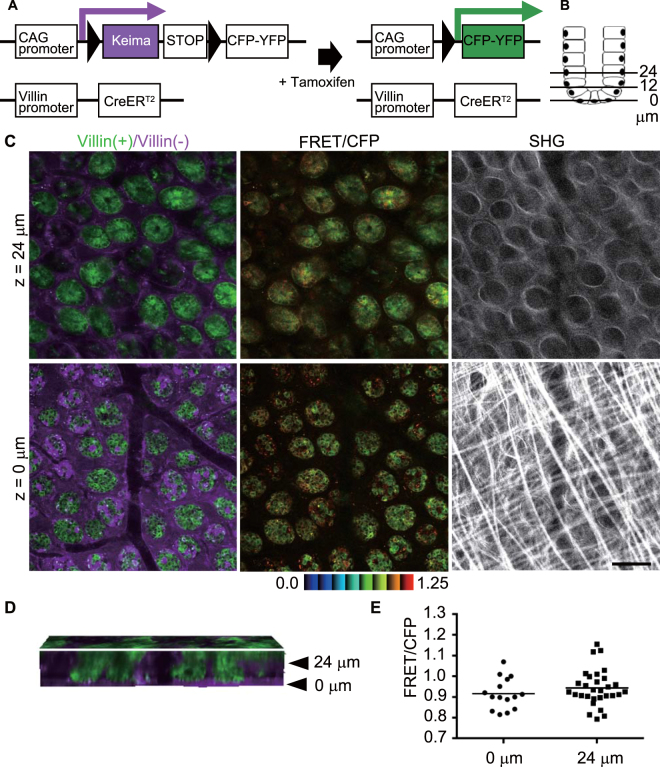
Establishment of Villin-Eisuke mice and TPM imaging of Erk activity in the normal ileum. (**A**) Schematic view of a Cre-lox system for intestinal epithelial cell-specific biosensor expression. (**B**) Schematic view of the small intestinal crypt. Epithelial cells at the crypt bottom were set to the z = 0 μm position, and the distance from the crypt bottom to the lumen is shown as 12 μm or 24 μm. (**C**) Representative images of a biosensor [green, denoted as Villin(+)] and the Keima protein [magenta, denoted as Villin(−)] of the normal intestine after recombination; z = 0 μm and z = 24 μm indicate the localization of the images as in Fig. 1B. The FRET/CFP ratio image is shown in intensity-modulated display mode (IMD) with a 32-intensity in 8-ratio. The upper and lower limits of the ratio range are also shown at the bottom of the panel. Second harmonic generation (SHG) images were taken for the same area with a different set of filters. Scale bar, 50 μm. (**D**) The cross sectional view of three-dimensional reconstruction of 75 images taken at 1 μm intervals (Movie 1). (**E**) Comparison of the FRET/CFP ratio between cells at the crypt bottom and those at z = 24 μm (*p* < 0.05; *t*-test). The graph shows the representative results from 3 independent experiments.

First, Eisuke mice, which carry a FRET biosensor for Erk^13^, were observed in order to monitor the Erk activity in the repair process in the ileum (Supplementary Fig. S1, Movie 1). The epithelial structure was disrupted by ischemia, and stromal cells, such as neutrophils, macrophages, and fibroblasts, were accumulated at the ischemic site. It was, therefore, difficult to identify the epithelial cells by their morphologies. To exclusively express FRET biosensors in intestinal epithelial cells, we used *Villin-CreERT2* transgenic mice^16^, which express a Cre recombinase in the intestinal epithelial cells, and *Lox-STOP-lox-Eisuke* mice^17^ to obtain a double compound mutant mouse carrying both *Villin-CreERT2* and *Lox-STOP-lox-Eisuke* (hereafter Villin-Eisuke). Villin-Eisuke mice expressed a Keima fluorescent protein in the whole tissue. Once Cre activation was achieved by administration of tamoxifen, the STOP sequence and Keima DNA, which are flanked by the lox sequence, were removed, resulting in a biosensor expression (Fig. 1A). Similarly, when *Lox-STOP-lox-PKA* mice were used, Villin-PKA-chew was generated.

Throughout the study, we set the cell at the base of the crypt to z = 0 μm and observed cells along the Z-axis toward the luminal side up to z = 24 μm (Fig. 1B). When observed with TPM from the serosal side, in the normal crypt, the majority of the epithelial cells were switched to express the biosensor, while epithelial cells without Cre activation and stromal cells both expressed Keima protein [Fig. 1C, left column, denoted as Villlin(+)/Villin(−)]. The FRET/CFP of the Erk biosensor in the crypt was comparable between cells in the crypt bottom and cells 24 μm from the crypt bottom. Collagen fibers detected by second harmonic generation (SHG) appeared in a reticular pattern with longitudinal fibers at the z = 0 level, while at the z = 24 level, they appeared as circular patterns corresponding to the basement membrane surrounding the crypt. The cross-section view of the three-dimensional reconstruction image showed that the epithelial cells were positioned vertically, forming the crypt, between z = 0 and z = 24 μm (Fig. 1D, Movie 2). To quantify Erk activity, cells in the crypt at each level were randomly selected in the CFP images and analysed for their Erk activity by calculating the FRET/CFP ratio. As shown in Fig. 1C, there was no significant difference of Erk activity between the levels in the crypts (Fig. 1E).

### Higher activity of Erk in resealing epithelial cells

**Figure 2:**
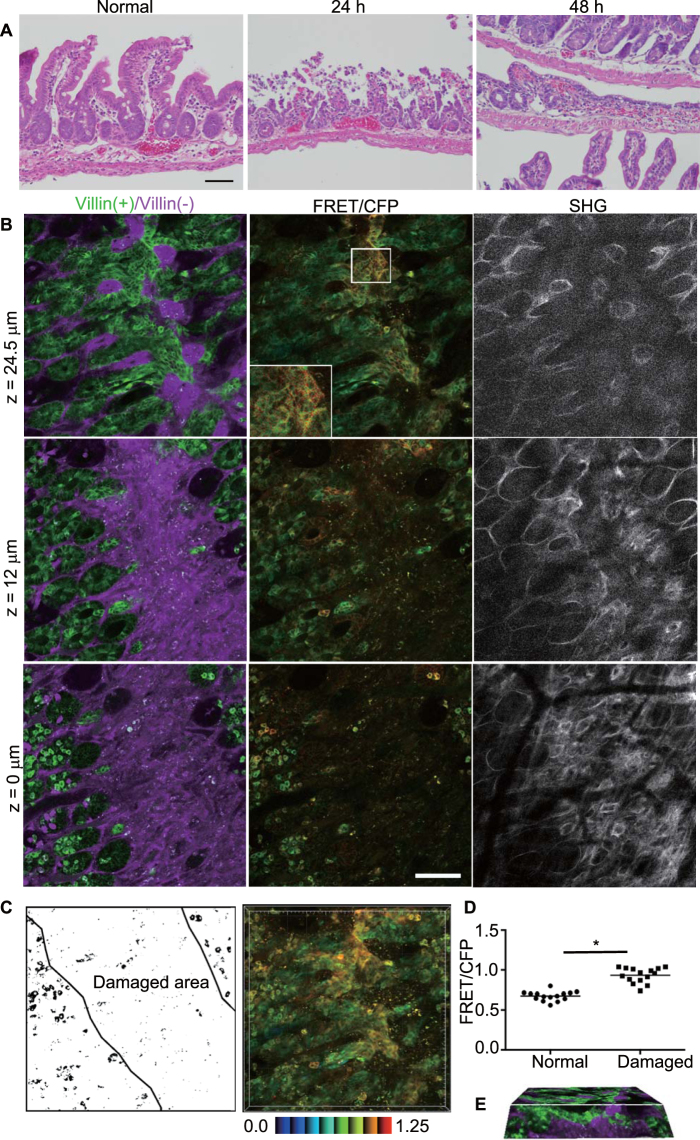
Activation of Erk in the resealing epithelial cells in the ileum. (**A**) H&E staining images of the normal mouse ileum (left), and the damaged ileal epithelia at 24 h (middle) or 48 h (right) after ischemia. Bar, 50 μm. (**B**) Representative images of the damaged ileum 48 h after ischemia. A biosensor [green, denoted as Villin(+)] and the Keima protein [magenta, denoted as Villin(−)] and FRET/CFP and SHG images are depicted as described in Fig. 1B. (C) The schematic view of the damaged area and the three-dimensional projection image using FRET/CFP images. The colour bar indicates the FRET/CFP ratio in IMD with the lower and upper limits of the ratio range. (**D**) Comparison of the FRET/CFP ratio between cells in normal crypts (denoted as Normal) and resealing epithelial cells (denoted as Damaged) at z = 24 μm (*p* < 0.05; *t*-test). The graph shows the representative results from 9 independent experiments. The average FRET/CFP values for the 9 experiments were 0.714 (for cells in normal crypts) and 0.835 (resealing epithelial cells). (**E**) The cross sectional view of the three-dimensional reconstruction by 75 images taken at 1-μm intervals (Movie 2).

To model ischemia injury, we employed segmental vascular occlusion, since it induces local infarction without severe damage to the other organs, and the lesion can be relatively easily detected in living mice. One of the mesenteric arteries near the cecum of a C57/B6 mouse was occluded to block the blood supply for 50–60 min (Supplementary Fig. S2). After reperfusion, the intestine was returned to the abdomen, and the wound was closed. In the absence of ischemia surgery, the intestinal mucosa was covered by columnar epithelial cells that formed a crypt-villus structure, as revealed by hematoxylin & eosin staining (Fig. 2A and Supplementary Fig. S3). Twenty-four hours after ischemia, the epithelial cells were detached from the basement membrane and the crypt-villus structure was disrupted, while the muscle layer was not damaged. Forty-eight hours after ischemia, monolayer epithelial cells appeared to cover the injured area. Therefore we imaged the intestine 48 h after ischemia surgery in this study.

In the Villin-Eisuke intestine, the FRET biosensor-expressing epithelial cells were not observed in the damaged area at the z = 0 level (Fig. 2B), whereas Keima-expressing stromal cells were abundant. At the higher levels, such as z = 12 or 24.5 μm, the number of FRET biosensor-expressing epithelial cells connecting to the neighbouring crypts was increased. The collagen fibers surrounding crypts became much denser than in the normal area, and the structure of the crypts was disorganized (Fig. 2B, SHG). From these images, we designated this area as the damaged area (Fig. 2C). Interestingly, higher Erk activity was observed in resealing epithelial cells in the damaged area compared to adjoining normal cells. When a series of Z-axial images were stacked into one plane, higher Erk activity at the centre of the damaged area was apparent (Fig. 2C). The FRET/CFP value of the cells in the crypt at z = 0 μm was comparable to that at z = 0 μm in the normal area (Fig. 2D). In the representative experiment, at z = 24.5 μm, the mean FRET/CFP value of resealing epithelial cells was 0.93, which was significantly higher than that of cells in the adjoining normal crypts (0.67) at the same z level (*p* < 0.05). To rule out the possibility that the higher FRET/CFP ratio in the injured area was due to an imaging artefact, Villin-PKA-chew was similarly imaged, revealing that PKA was suppressed in the resealing epithelial cells (Supplementary Fig. S4). Three-dimensional reconstruction of a series of Z-axis images confirmed that epithelial cells were elongated horizontally from adjacent normal crypts to cover the damaged area (Fig. 2E, Movie 3).

### Yap-Egfr-Erk axis in the covering epithelial cells

**Figure 3:**
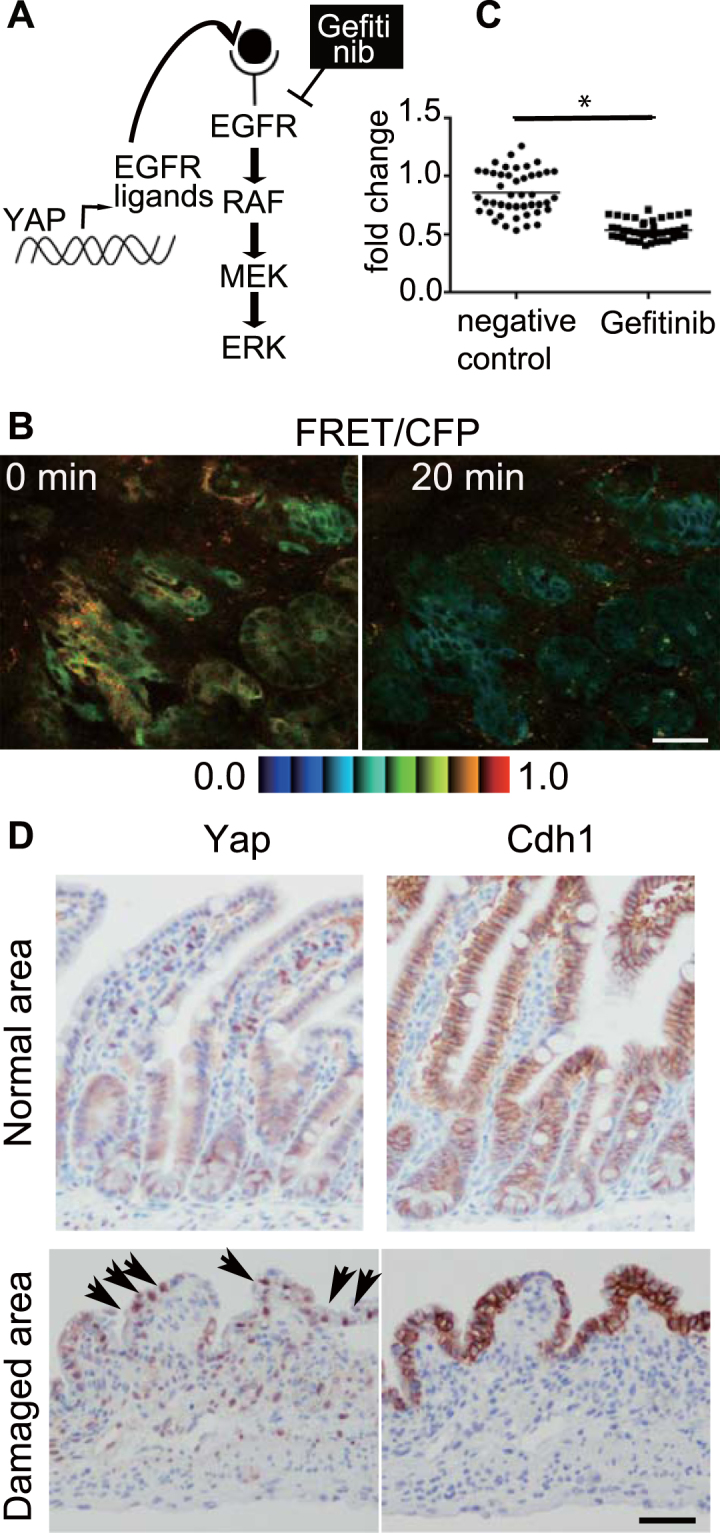
Yap-Egfr-Erk axis in the resealing epithelial cells. (**A**) A schematic view of the Yap-Egfr-Erk signalling pathway and Gefitinib, an Egfr inhibitor. (**B**) A representative image of the FRET/CFP ratio of the resealing epithelial cells before (0 min) and after (20 min) Gefitinib treatment. The lower and upper limits of the FRET/CFP range are expressed with an IMD colour bar. Scale bar, 50 μm. (**C**) Three independent experiments were performed, and 13–15 cells were analysed in each experiment. The FRET/CFP ratio of individual cells at 20 min after treatment with solvent (denoted as a negative control) or Gefinitib was normalized to the FRET/CFP ratio before treatment (*p* < 0.0001, unpaired *t*-test). (**D**) The immunohistochemical images of Yap and Cdh1 in the normal ileum (upper panels) and 48 h after the ischemia (lower panels). Arrows indicate cells with Yap nuclear translocation. The images are representative of the 3 independent experiments. Bar, 100 μm.

Erk is a threonine/tyrosine kinase located downstream of various extracellular growth factors. One of the major upstream pathways is the epidermal growth factor receptor (Egfr)-Grb2-SOS-Ras-Raf pathway^18,19^ (Fig. 3A). To examine whether higher Erk activity in resealing epithelial cells is regulated by Egfr, Gefitinib, a selective inhibitor which disrupts Egfr kinase activity by reversibly binding within the ATP-binding pocket of the Egfr protein^20^, was administrated intravenously. The higher FRET/CFP ratio in resealing epithelial cells (Fig. 3B, 0 min) was decreased by Gefitinib treatment (Fig. 3B, 20 min and Supplementary Fig. S5). Quantification of individual cells confirmed the decrease in the FRET/CFP ratio (Fig. 3C and Supplementary Fig. S6A). Administration of solvent (0.4% lactic acid/water) slightly altered the FRET/CFP ratio (68–106%, Supplementary Fig. S6B) without significance. To confirm that the degree of decrease upon addition of solvent and Gefitinib was significant, we calculated the FRET/CFP ratio, which was normalized to that before treatment (Fig. 3C, the right graph). The results indicated that Erk activation in the resealing epithelial cells was dependent on Egfr activation.

It has been reported recently that Egfr-Erk pathway activation is mediated via upregulation of ligands for the Egf receptor by transcriptional activation of Yap in the regeneration process of intestinal epithelial cells^21^ (Fig. 3A). Immunohistochemical analysis revealed Yap translocation in the resealing epithelial cells, whereas cytoplasmic localization of Yap was dominant in the normal crypts (Fig. 3D). Cdh-1 immunostaining confirmed that a monolayer of epithelial cells covered the injured area. Similar Yap localization to the nucleus was observed 24 h after ischemia (Supplementary Fig. S7). These data suggest that Erk activation in the covering epithelial cells involves Yap translocation.

### Cell cycle analysis during the regeneration

**Figure 4:**
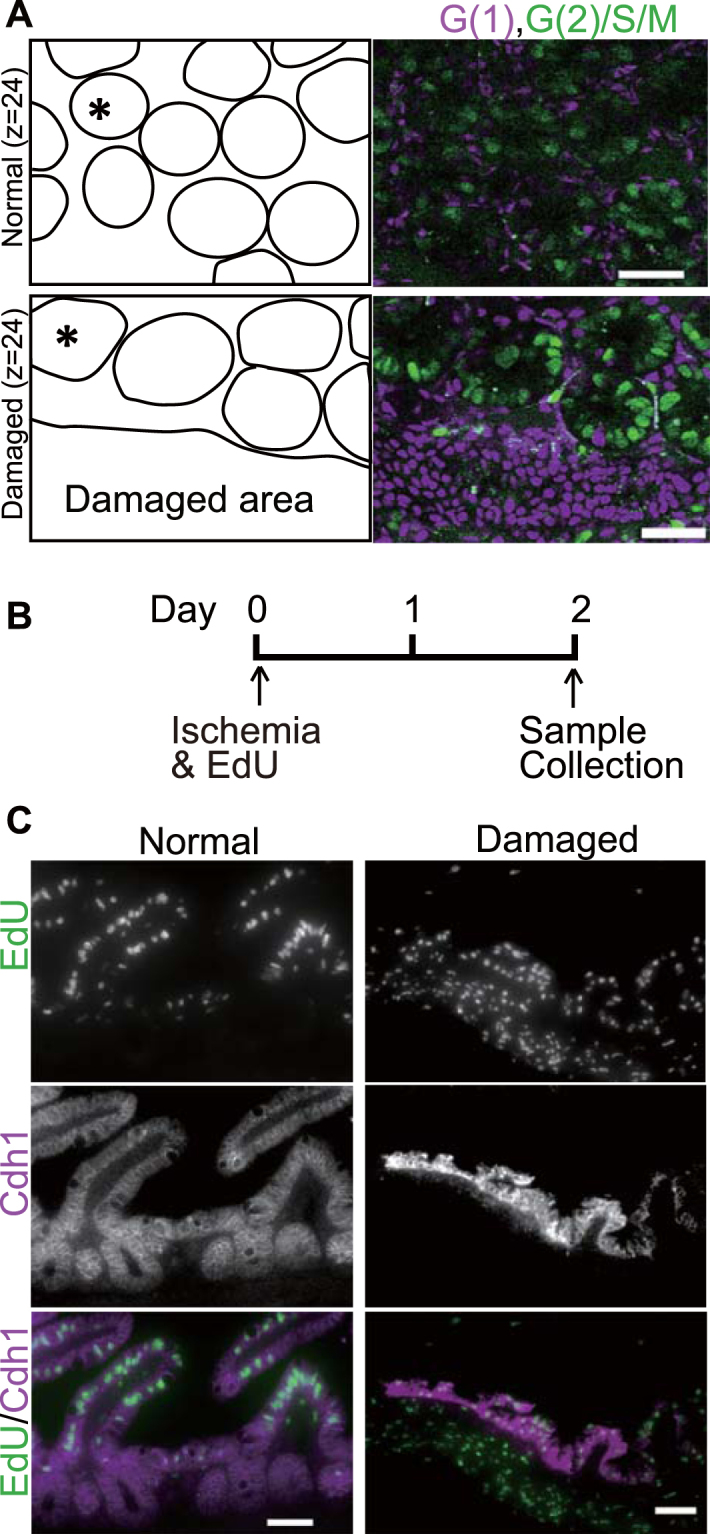
Cell cycle and proliferation analysis during the regeneration after injury. (**A**) The ileum of a R26p-Fucci2 transgenic mouse without injury (Normal, the upper panels) and that 48 h after ischemia (Damaged, the lower panels) at Z = 24 μm were imaged by TPM. A schematic view of the location of the crypts (one of the crypts is indicated with an asterisk (*)) and the damaged area is given in the left panels. Cells in G_1_ and G_2_/S/M phase are shown in magenta and green, respectively. The images shown are representative of 4 independent experiments. Bars, 50 μm. (**B**) The experimental design for analysing proliferation after the injury. Mice aged 7 to 15 weeks were injected with EdU immediately after the ischemia surgery. Mice were sacrificed 2 days after the ischemia for histology. (**C**) Fluorescent images of EdU labelling and anti-Cdh1 antibodies from the mouse without ischemia (denoted as Normal), and that with ischemia (Damaged). Bars, 50 μm. The images shown are representative of 3 independent experiments.

Since Erk is involved in cell proliferation^18^ and important for cell cycle progression^22^, the next question to be addressed was whether resealing epithelial cells proliferate. To answer this question, cells of *R26p-Fucci2* transgenic mice, which carry a cell cycle indicator, Fucci, in a single transgene driven by the Rosa26 promoter^23^, were imaged. In the normal region, at z = 24 μm, most of the epithelial cells in the crypt were in S/G_2_/M phase, as shown in green (Fig. 4A). These cells were likely to be transit-amplifying cells, which divide every 24 h^24^, while the spindle-shaped cells surrounding crypts were in G_1_ phase. In the damaged area, the covering epithelial cells, which are usually observed at z = 24 μm under our observation settings, were in the G_1_ phase, suggesting that covering epithelial cells did not proliferate.

To determine when these resealing epithelial cells were generated, EdU was administrated to label the newly synthesized cells immediately after ischemia (Fig. 4B). Forty-eight hours later, the intestine was collected and EdU-positive cells were visualized. In the normal area, where proliferating cells in the crypt migrate up to the tip of the villi within 2 days, EdU-labelled cells were no longer located in the crypt, but rather in the middle of the villus. On the other hand, epithelial cells covering the damaged area, which appeared as Cdh1-positive cells, were EdU-positive, indicating that resealing epithelial cells were generated after the injury.

### ERK activation in human intestinal ulcer specimens

**Figure 5:**
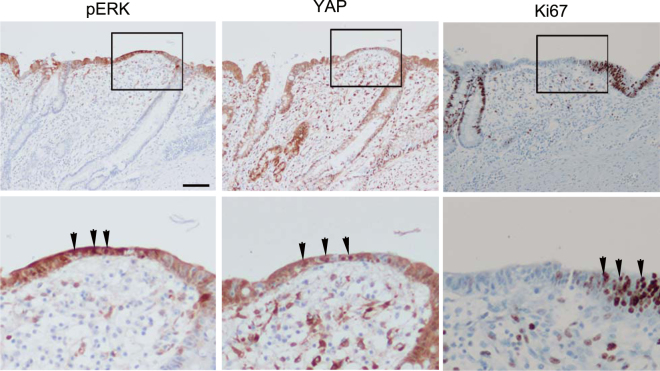
Immunohistochemical analysis of human intestinal ulcers. A representative image (patient1-lesion1) of anti- pERK, YAP, and Ki67 in a human intestinal ulcer. The lower panels are higher magnification images of the squares in the upper panels. Arrows indicate the positive cells with antibodies. Bar, 100 μm.

To confirm that our findings are relevant to the epithelial resealing process in humans, specimens from the resected intestine with ulcer(s) were stained with antibodies. Five lesions were positive for phospho-ERK (pERK) staining of resealing epithelial cells, whereas no or weak staining was observed in the epithelial cells forming a crypt-like structure (Table 1 and Fig. 5). In three out of five samples with pERK nuclear staining, YAP nuclear translocation was observed in the subpopulation of resealing epithelial cells where pERK nuclear staining was observed. These results suggest that the YAP-EGFR-ERK signalling axis is involved in the monolayer of epithelial cell resealing. In addition, the majority of pERK-positive resealing epithelial cells were Ki67-negative, consistent with the results obtained from the mouse model (Fig. 4). It is interesting that, in the ulcer region, YAP nuclear translocation was also frequently observed in the epithelial cells without pERK, and yet these cells were also Ki67-positive, suggesting that YAP activation may contribute to cell proliferation in the regeneration process.

**Table 1:** Summary of pERK, YAP and Ki67 in human ulcers.

Patient-lesion	pERK^1^	nYAP^2^	Ki67	Location	Age, year	Sex
1-1	(+)	(−/+)	(−)	Jejunum	80	Male
1-2	(+)	(−/+)	(−)	Jejunum	80	Male
2-1	(+)	(−)	(−)	Ileum	43	Female
3-1	(+)	(−)	(−)	Ileum	0 (3 month)	Male
4-1	(+)	(+)	(−/+)	Ileum	45	Male

^1^phospho ERK.

^2^nuclear YAP.

## Discussion

**Figure 6:**
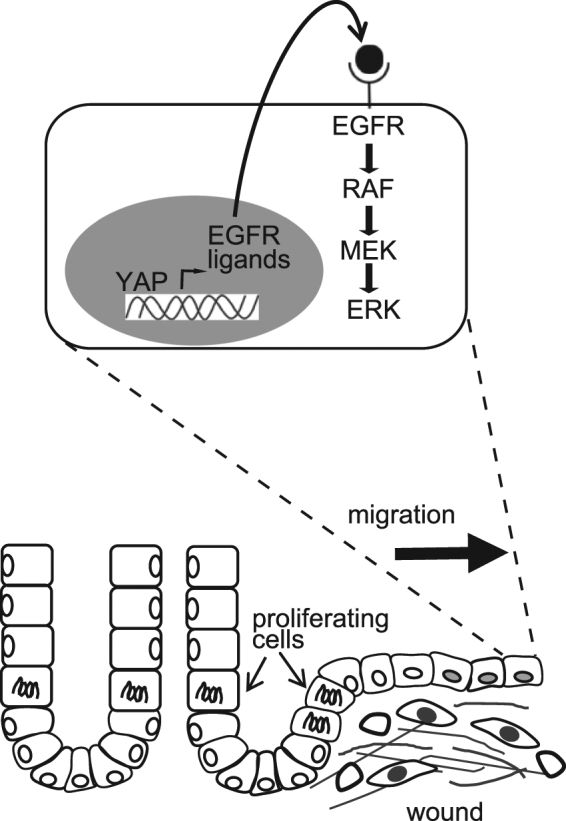
Model of the resealing epithelial cells.

To our knowledge, this is the first report to observe Erk activation in the epithelial cells covering an area of ischemic injury in living mice. Since an Egfr inhibitor suppressed the Erk activation (Fig. 3), it was indicated that an Egfr-Erk signalling pathway was involved in the epithelial cell resealing process (Fig. 6). The source of Egfr ligands might be epithelial cells, since a study on the ileum tissues from patients with Crohn’s disease showed that ulceration of the epithelium induced the formation of new cell lineages which did not proliferate but secreted EGF^25^. Considering that the resealing epithelial cells in our model also did not proliferate (Fig. 4), it is possible that they secreted Egf to stimulate Egfr-Erk signalling in an autocrine manner. In support of this possibility, we detected Yap nuclear localization in resealing epithelial cells both in mice and humans (Figs 3 and 5). In addition, it has been reported that in irradiated mice, Yap is translocated to the nucleus, which in turn induces transcription of Egfr ligands, such as *Areg* ^21^. In that study, the authors deleted Yap in Lgr5-positive stem cells, and found that the Yap-deleted epithelial cells could not survive after irradiation. In our study, the resealing epithelial cells were not stem cells, because they were produced just after ischemia (Fig. 4). Despite the many differences between stem cells and non-stem cells, we assume that once Yap is located to the nucleus, transcriptional activation is induced to produce the common growth factors. In our preliminary immunostaining experiments, specific Areg expression was not detected in the injured area (data not shown), suggesting other ligands might be involved in the covering epithelial cells. Among the various upstream factors of Yap, we assume that secretion of cytokines such as interleukin (IL)-6 and IL-11 might be involved, since Yap nuclear translocation can be stimulated by gp130-YAP signalling in the intestine^26^, and IL-6 is elevated upon ischemia and reperfusion of arteries in the human rectum^27^. It has been reported that the Notch pathway is involved in signalling pathway triggered by gp130^26^. In addition, the PGE_2_-YAP axis was recently shown to be important in the colon-regeneration process^28^. We detected COX-2 (a rate-limiting enzyme in the process of PGE2 synthesis)-expressing stromal cells accumulated near the covering epithelial cells 48 h after ischemia (data not shown). Therefore it is possible that PGE_2_ from stromal cells partly activates Yap for the epithelial cell regeneration.

Taking all these findings into consideration, we propose the following model (Fig. 6): in response to ischemia-induced injury, epithelial cells in the crypt adjoining the injured area start to proliferate within several hours, and cells generated during that period migrate to cover the injured area. It has been established previously that rapid (within several hours) resealing of the epithelial barrier following injuries is accomplished by a process termed epithelial restitution^1,29^. To complete the resealing of the injured area, it is thought that additional delayed mechanisms of epithelial repair play a role, including increased epithelial cell and epithelial cell differentiation. In the biopsy-wounded colon, a specialized type of repair cells, named wound associated epithelial cells (WAE), have been characterized^4,5,30^. Despite the difference of injury (ischemia vs. biopsy), site (small vs. large intestines), and timing (day 2 vs. days 2–4) between our study and these earlier ones, the epithelial cells covering the injured area share common features—namely, they form a monolayer of cuboidal morphology without proliferation. Further experiments will be required to conclude that they utilize the same molecular mechanisms. Since methods for the stem cell/organoid-engraftment to the small and large intestinal mucosa have now been established^31,32^, knowledge of the molecular mechanisms by which migrating epithelial cells form the complete mucosa will accelerate digestive tract-regeneration therapy.

We still lack direct evidence that these epithelial cells migrate, even though we were able to live-image the kinase activity in the cells. We previously imaged the fast-migration and extravasation processes of neutrophils in lipopolysaccharide-treated intestine^33^. The difference in velocity between our present and previous studies is probably dependent on the cell types, microenvironment and/or whether single cells or cell clusters were studied. Further techniques for imaging migrating cells *in vivo*—including long-term *in vivo* imaging and the collection of parameters such as cellular kinase activities, velocity, and direction—will assist in the development of effective reagents to treat inflammation, various types of cancer, and other pathological conditions without side effects.

## Materials and Methods

### Mice

Mice were housed in a specific pathogen-free facility under a 12-h light-dark cycle and received a routine chow diet and water ad libitum. lox-Eisuke (nbio213), R26p-Fucci2 (CDB0203T), and *Villin-CreER* ^*T2*^ transgenic mice were obtained from the National Institute of Biomedical Innovation (Osaka, Japan)^17^, RIKEN (Saitama, Japan)^23^, and Dr. Sylvie Robine (Curie Institute, Paris)^16^, respectively. Tamoxifen (Sigma-Aldrich Japan Inc., Tokyo) was dissolved in corn oil (Sigma-Aldrich), and 2 mg per mouse was administrated by intraperitoneal injection (i.p.) for 3 days to induce Cre activity in mice at 5–8 weeks of age.

### Ischemia surgery and *in vivo* imaging

One to eight weeks after tamoxifen administration, mice were anesthetized by 2,2,2-tribromoethanol (Sigma-Aldrich) at a dose of 0.4 g/kg before ischemia surgery. The period after tamoxifen treatment in this study is summarized in Supplementary Table S8. The abdominal area of the mouse was disinfected using 70% ethanol. A small vertical incision was made in the middle of the abdominal wall. The middle part of the intestine was exteriorized and one of the mesenteric arteries located in the ileum (~3 cm above from the cecum, Supplementary Fig. S2) was occluded by micro clips for 50–60 min. After the release of the clip, the intestine was carefully returned to its anatomical position. The abdomen wound was closed with surgical clips. Twenty-four or 48 h after the ischemia surgery, mice were anesthetized with 0.5–2% isoflurane (Wako Pure Chemical Industries, Ltd., Osaka, Japan) inhalation at 1.0 L/min by a small animal anesthetizer (Biomachinery, Chiba, Japan), then laid on a heat plate (TOKAI HIT, Shizuoka, Japan) at 37 °C. The intestine was exteriorized from the peritoneal cavity, and the oral- and tail-side of the ischemia area (around 3–5 cm in length) were clipped. The lumen of the clipped intestine was filled with phosphate buffered saline [PBS(−)] with a 27-gauge needle. The small intestine was then fixed with an aspiration fixation system^11^ for *in vivo* observation. For inhibitor experiments, Gefitinib (Santa Cruz Biotechnology, CA) was injected at 25 mg/kg with 10 μl of Q-Tracker 655 (Thermo Fisher Scientific, MA) into the intra-orbital space or jugular vein, and the intestine was imaged for 40 min. All animal experiments were approved by the Institutional Animal Research Committee of Kanazawa Medical University.

### Two-photon excitation microscopy and image processing

Two-photon microscopy was performed with an FV1200MPE-BX61WI upright microscope (Olympus, Tokyo) equipped with a 25x/1.05 water immersion objective lens (XLPLN 25XWMP; Olympus) and an InSight DeepSee Ultrafast laser (Spectra-Physics, Santa Clara, CA). Three dichroic mirrors, DM450, SDM505 and SDM570 (Olympus), were used. Emission filters were purchased from Olympus; BA647–57 was used for the detection of Keima, BA 425/26 for second harmonic generation (SHG), BA520-560 for YFP and BA460-500 for CFP. For FRET imaging an 840 nm excitation wavelength was used as described previously^33^. For Fucci imaging, wavelengths of 960 nm and 1030 nm were used to excite Venus and mCherry proteins, respectively. The dichroic mirrors were SDM505 and SDM570. The emission filters were BA472/30 for the detection of second harmonic generation (SHG), BA495-540 for Venus, and BA575-630 for mCherry. Images were acquired using an FV10-ASW viewer (Olympus) with 512 × 512 image size and 12.5 μs/pixel scan speed.

Acquired images were analysed with MetaMorph software (Universal Imaging, West Chester, PA) as described previously^34^. In brief, the level of FRET was represented by the FRET/CFP ratio image in intensity modulated display mode; eight colours from red to blue are used to represent the FRET/CFP ratio, and the 32 grades of colour intensity are used to represent the signal intensity of the CFP image. The warm and cold colours indicate high and low FRET levels, respectively. A series of Z-stack images with a 1 μm interval was reconstituted into the three-dimensional structure by Imaris software (Bitplane, Switzerland).

### Statistics

Student’s *t*-test was performed and dot plots were generated using Prism6 software (GraphPad).

### Histology

Twenty-four or 48 h after ischemia, mice were euthanized with CO_2_ and their necks were dislocated. The small intestines were collected, washed with PBS(−) several times, fixed in Mildform (Wako Pure Chemicals), and embedded in paraffin. Four μm-thick slices were deparaffinized and stained with hematoxylin and eosin. Immunohistochemistry was performed by a standard protocol. Briefly, sections were boiled in a Pascal pressure cooker (Dako Japan, Tokyo) for 10 min at 100 °C in 10 mM citrate buffer (pH 6.0), then left for 30 min at room temperature. After treatment with 3% H_2_O_2_ in PBS(−), the sections were incubated with primary antibodies against Yap [Cell Signalling Technology (CST), Beverly, MA] or Cdh1 (CST), which were diluted 1:400 in SignalStain Antibody Diluent (CST), for 1 h at room temperature. For the detection of phospho-Erk1/2 (Thr202/Tyr204 of Erk1 and Thr185/Tyr187 of Erk2), the primary antibody (CST) was diluted at 1:200 followed by incubation overnight at 4 °C. Primary antibodies were labelled with a peroxidase using Histofine simple stain (Nichirei Bioscience Inc., Tokyo, Japan). Signals were visualized with a DAB substrate kit (Dako).

### EdU labelling and detection

A Click-iT EdU Alexa Fluor 488 Imaging Kit (Thermo Fisher Scientific) was used according to the manufacturer’s protocol. Briefly, mice were injected with 100 μl of EdU (5-ethynyl-2′-deoxyuridine, 10 mg/ml) dissolved in DMSO by i.p., immediately after ischemia. Forty-eight hours later, the mice were euthanized, and the intestine was processed as described above (Histology section). Slices of 4-μm thickness were used for the EdU Click-iT reaction, and incubated with the anti-Cdh1 antibody followed by a secondary antibody against rabbit IgG conjugated with Alexa 596 (Thermo Fisher Scientific). Sections were mounted with Hard Set Mounting Medium with DAPI (Vector Laboratory Inc., CA) and scanned by NanoZoomer (Hamamatsu Photonics KK, Hamamatsu, Japan) or imaged by an IX81 inverted microscope (Olympus).

### Human specimens

The cases diagnosed with intestinal ulcers between 2011 and 2015 at Kanazawa Medical University Hospital were extracted from the surgical pathology files. Written informed consent was obtained from all patients enrolled in the study at the time of surgery, and the study was approved by the research ethics committee of Kanazawa Medical University Hospital. Formalin-fixed paraffin-embedded samples were cut at 4 μm thickness. For Ki67 IHC, a Ki67 antibody (Dako) and an automated staining instrument, BenchMark GX (Roche), were used according to the manufacturer’s protocol. Primary antibodies for YAP and pERK were as described above. To visualize the bound antibody, an EnVision Dual Link System-HRP (Dako) and DAB substrate kit were used. We also stained 6 lesions from 3 patients, who were operated on during 2006 and 2007, and found no positive signals of pERK (data not shown). Since we could detect YAP and Ki67 signals in the same sample, it was suspected that the phosphorylation of proteins was lost during long-term storage in paraffin. We therefore used the data obtained from recently acquired specimens in this study.

## Electronic supplementary material

Supplementary Information

Movie 1. Corresponding to Supplementary Figure S1

Movie 2. Corresponding to Figure 1D

Movie 3. Corresponding to Figure 2E
