# Supplementary material for: Activation of Erk in ileal epithelial cells engaged in ischemic-injury repair
Source: Sci Rep. 2017 Nov 28;7:16469. doi: 10.1038/s41598-017-16714-6 (PMC5705649; doi:10.1038/s41598-017-16714-6)
Supplement: Supplementary file 1 — Supplementary Information [file 41598_2017_16714_MOESM1_ESM.pdf]

**Supplementary Information**

# Activation of Erk in ileal epithelial cells engaged in ischemic-injury repair.

**Short running title: Erk activation during epithelial cell repair**

**Haruna Takeda and Etsuko Kiyokawa**

**Supplementary Figure S1.** FRET/CFP image of the injured ileum of Eisuke mice.

**Supplementary Figure S2.** Macroscopic view of a mesenteric artery occlusion for ischemia.

**Supplementary Figure S3.** H&E stained wide-field images of the damaged ileal epithelia at 24 h or 48 h after ischemia. (Corresponding to Figure 2A).

**Supplementary Figure S4.** Lower activity of PKA in resealing epithelial cells.

Supplementary Figure S5. The FRET/CFP of individual cells upon Gefitinib treatment (wide-field image of Figure 3B).

**Supplementary Figure S6.** The FRET/CFP of individual cells upon solvent and Gefitinib treatment (raw data of Figure 3C).

**Supplementary Figure S7.** Immunohistochemical images of Yap and Cdh1 at 24 h after ischemia (corresponding to Fig. 3D).

**Supplementary Table S8** The periods after tamoxifen treatment in this study.

**Movie 1. Corresponding to Supplementary Figure S1.**
Forty-eight hours after ischemia surgery, the damaged area in the ileum of an Eisuke mouse was imaged at 3-min intervals for 1 h.

**Movie 2. Corresponding to Figure 1D. 3D reconstruction of the ileum without**

**damage.**

After treatment with tamoxifen, the ileum of a Villin-Eisuke mouse was imaged by TPM. Images were taken at 1.0 μm intervals for 67 slices, and reconstituted with Imaris software.

**Movie 3. Corresponding to Figure 2E. 3D reconstruction of the ileum 48 h after ischemia surgery.**

After treatment with tamoxifen, Villin-Eisuke mice with ischemia surgery were imaged by TPM. Images were taken at 0.5 μm intervals for 144 slices, and reconstituted with Imaris software.

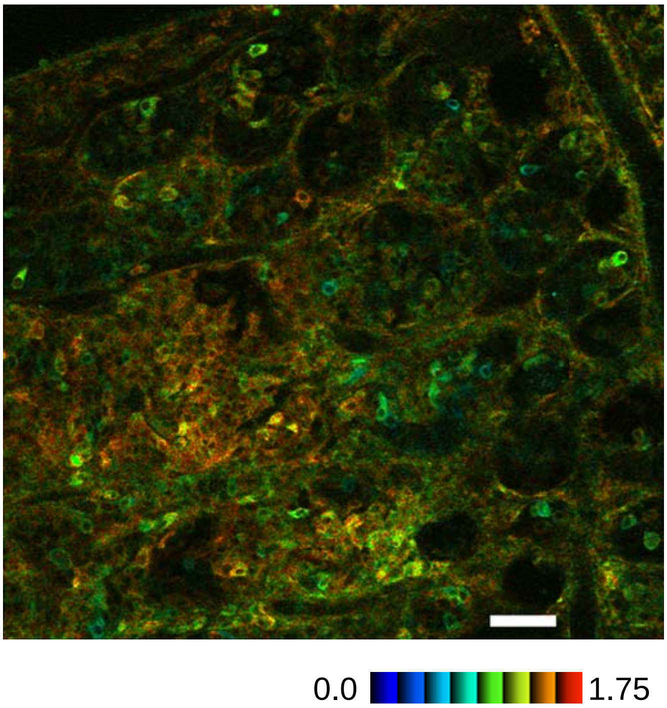


**Supplementary Figure S1.**
**FRET/CFP image of the injured ileum of Eisuke mice.**
Forty-eight hours after the ischemia surgery, the ileum of Eisuke mice was imaged by TPM. The FRET/CFP ratio image is shown in intensity-modulated display mode (IMD) with 32-intensity in 8-ratio. The upper and lower limits of the ratio range are also shown at the bottom of the panel. Bar, 50 μm. This image is taken from Movie 1.

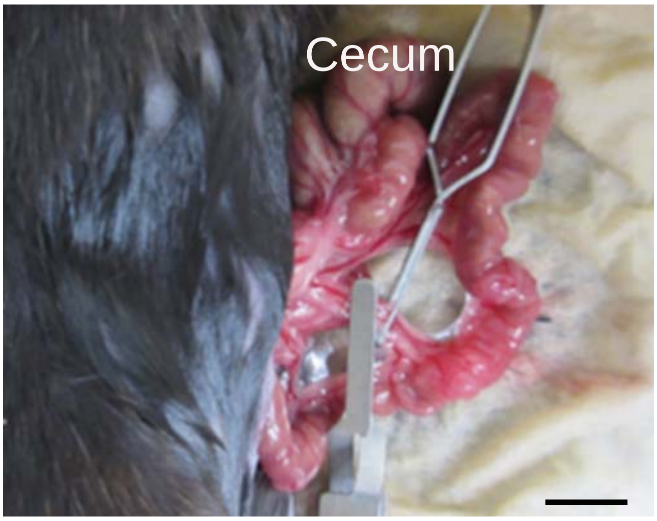


**Supplementary Figure S2.**
**Macroscopic view of a mesenteric artery occlusion for ischemia.**
The small intestine was pulled out from the peritoneal cavity of an anesthetized C57/B6 mouse. One of the mesenteric arteries near the cecum was occluded by clips to block the blood flow for 50-60 min. Scale bar, 5 mm.

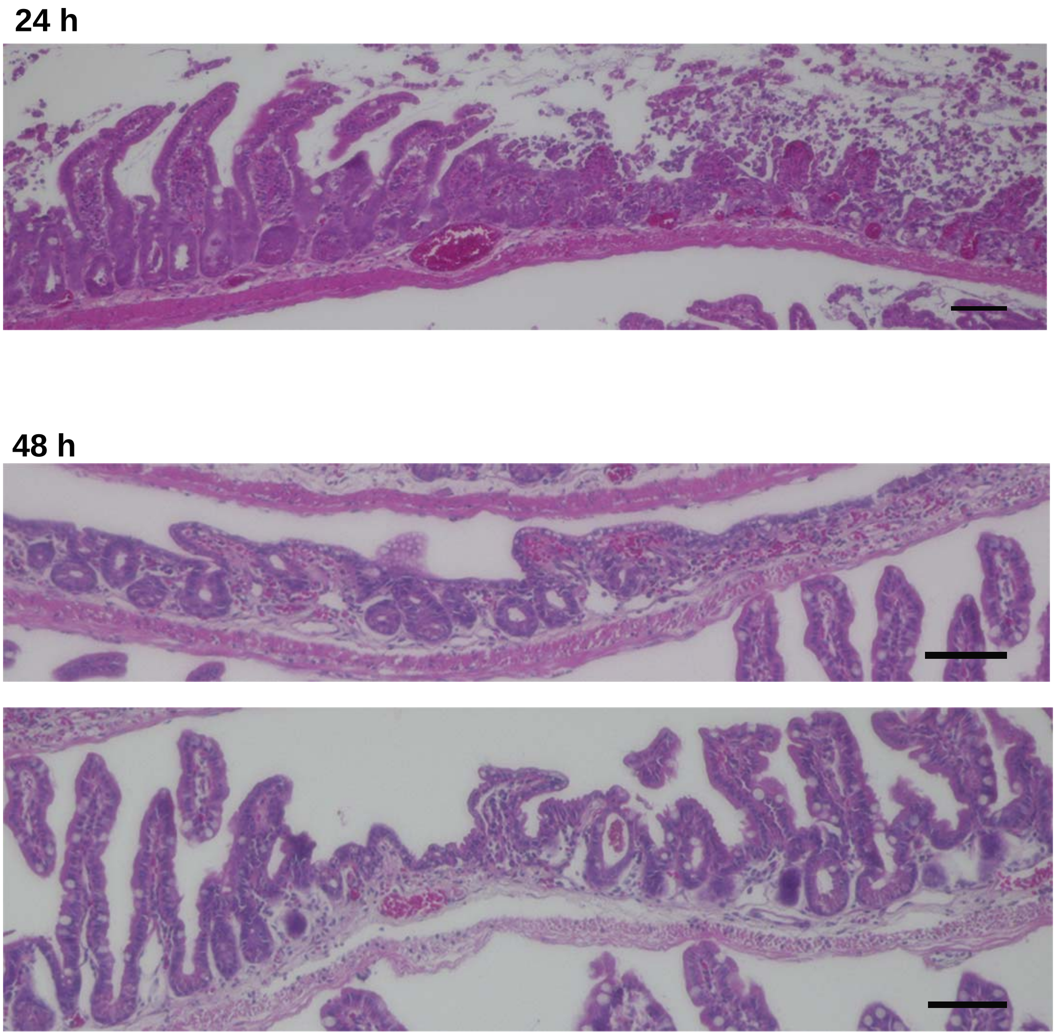


**Supplementary Figure S3.**
**H&E stained wide-field images of the damaged ileal epithelia at 24 h or 48 h after ischemia. (Corresponding to Figure 2A).**
Bar, 50 µm.

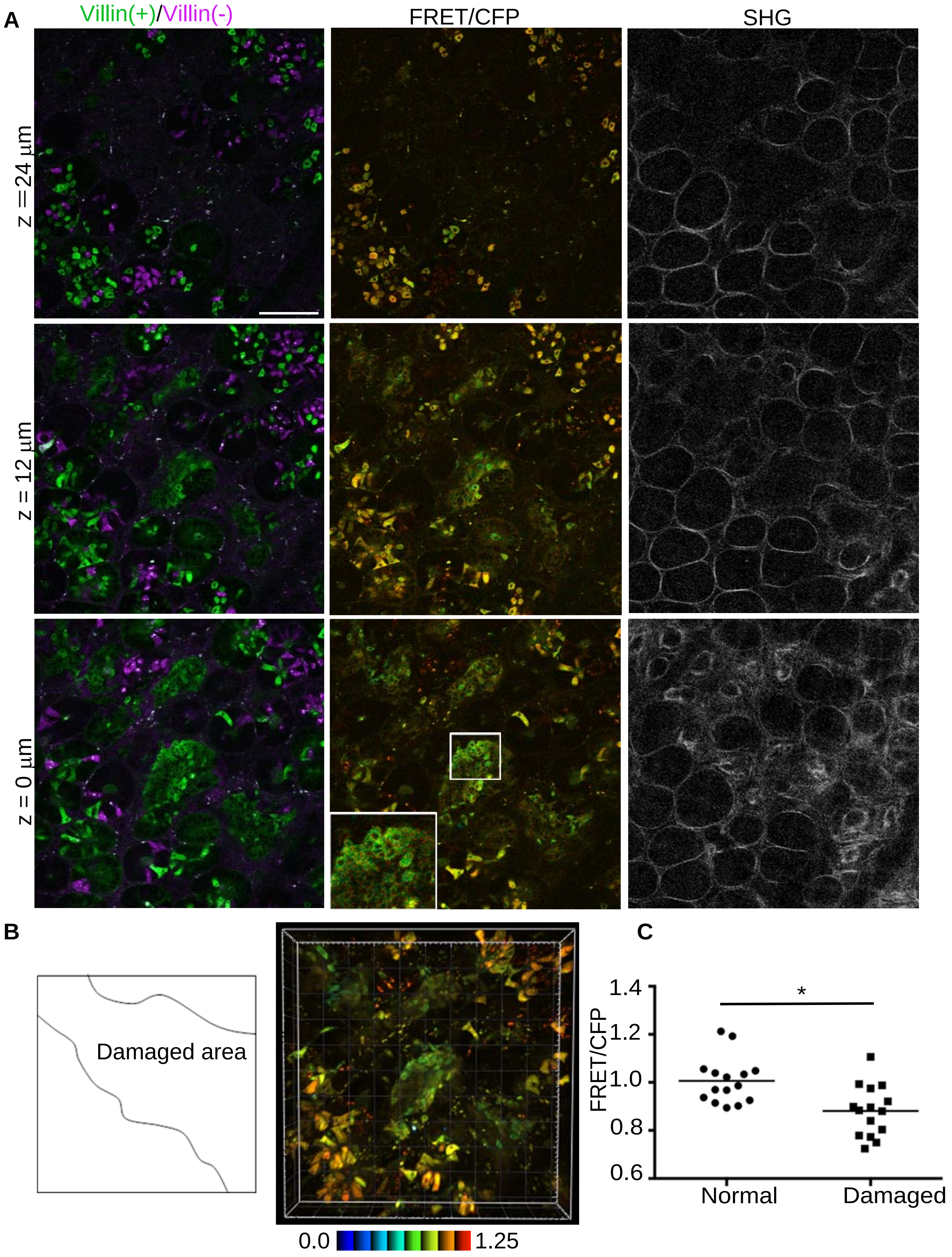


**Supplementary Figure S4. Lower activity of PKA in resealing epithelial cells.**
(A) Representative images of the damaged ileum of a Villin-PKA-chew mouse 48 h after ischemia. A biosensor [green, denoted as Villin(+)] and the Keima protein [magenta, denoted as Villin(-)] and FRET/CFP and SHG images are depicted as described in Figure 1B. (B) Schematic view of the damaged area and the three-dimensional projection image using FRET/CFP images. The colour bar indicates the FRET/CFP ratio in IMD with the lower and upper limits of the ratio range. Bar, 100 µm. (C) Comparison of the FRET/CFP ratio between cells in normal crypts (denoted as Normal) and resealing epithelial cells (denoted as Damaged) at z=24 µm ($p<0.05$; t-test). The graph shows the representative results from 3 independent experiments.

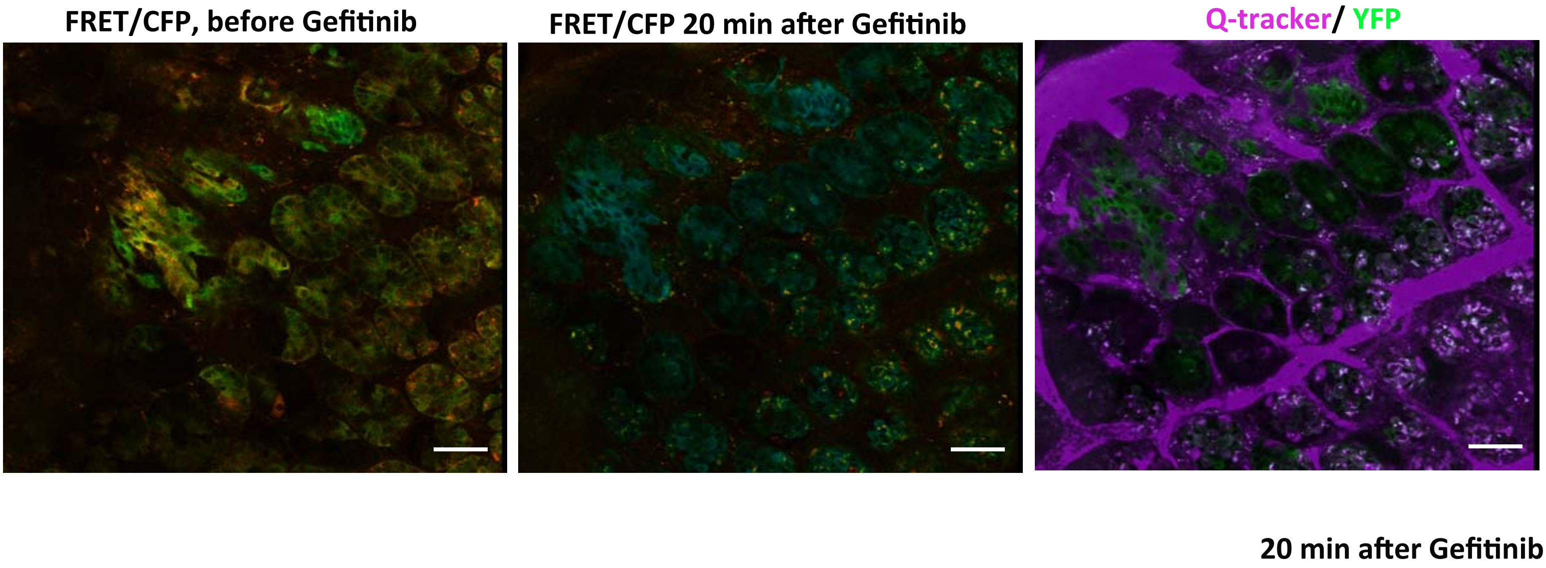


**Supplementary Figure S5.**
**The FRET/CFP of individual cells upon Gefitinib treatment (wide-field image of Figure 3B).**
A representative image of the FRET/CFP ratio of the resealing epithelial cells 20 minutes after Gefitinib treatment. In the right panel, images of YFP and RFP channels, which correspond to the biosensor and Q-tracker (venous flow and leaked area from the vein/capillary), respectively, were merged. Bar, 50 μm.

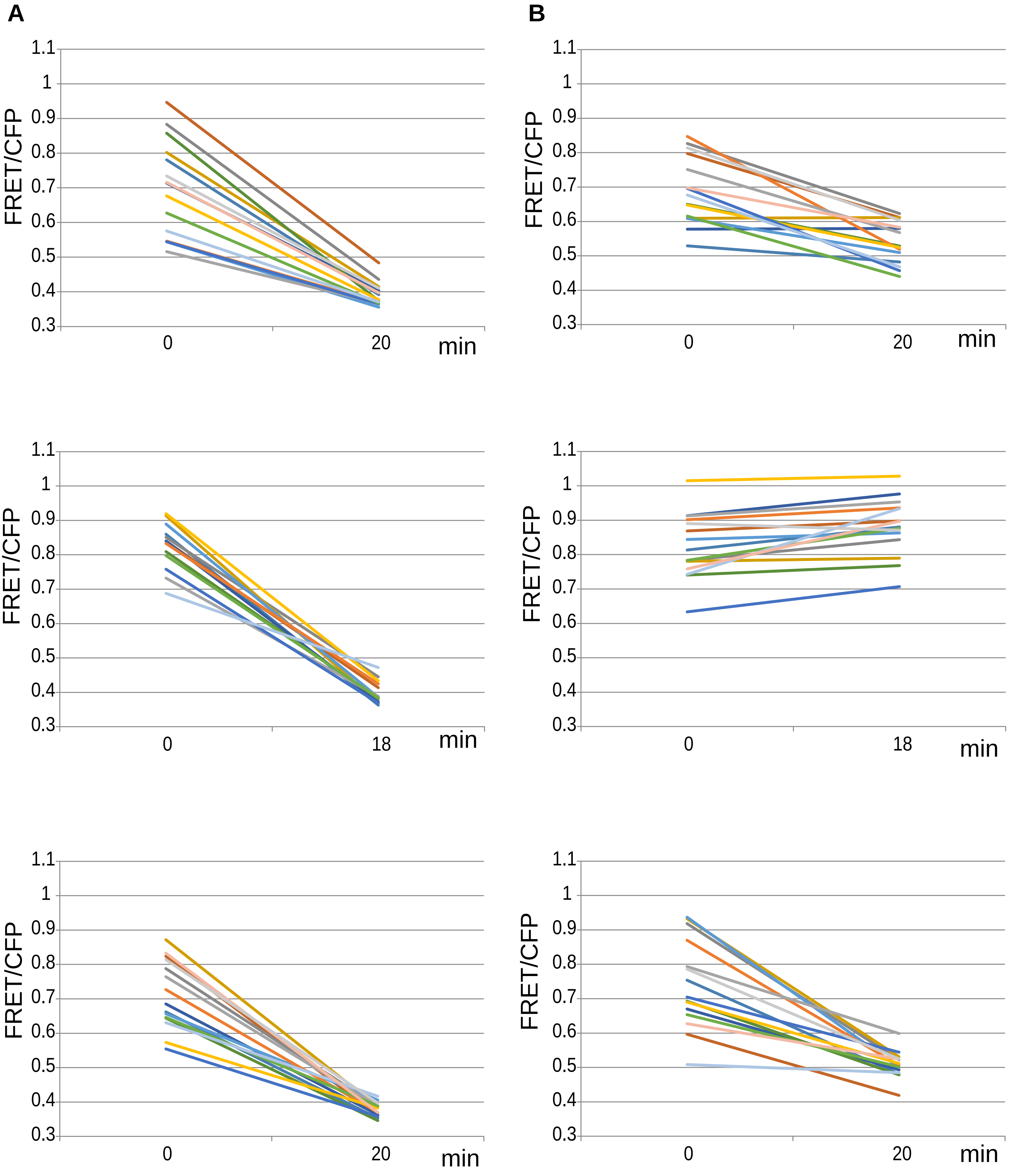


**Supplementary Figure S6**

**The FRET/CFP of individual cells upon solvent and Gefitinib treatment (raw data of Figure 3C).** Thirteen to fifteen cells in each experiment were analyzed. Forty-eight hours after ischemia surgery, Gefitinib (A) or water containing 0.4% lactic acid (B) was injected intravenously into injured Villin-Eisuke mice.

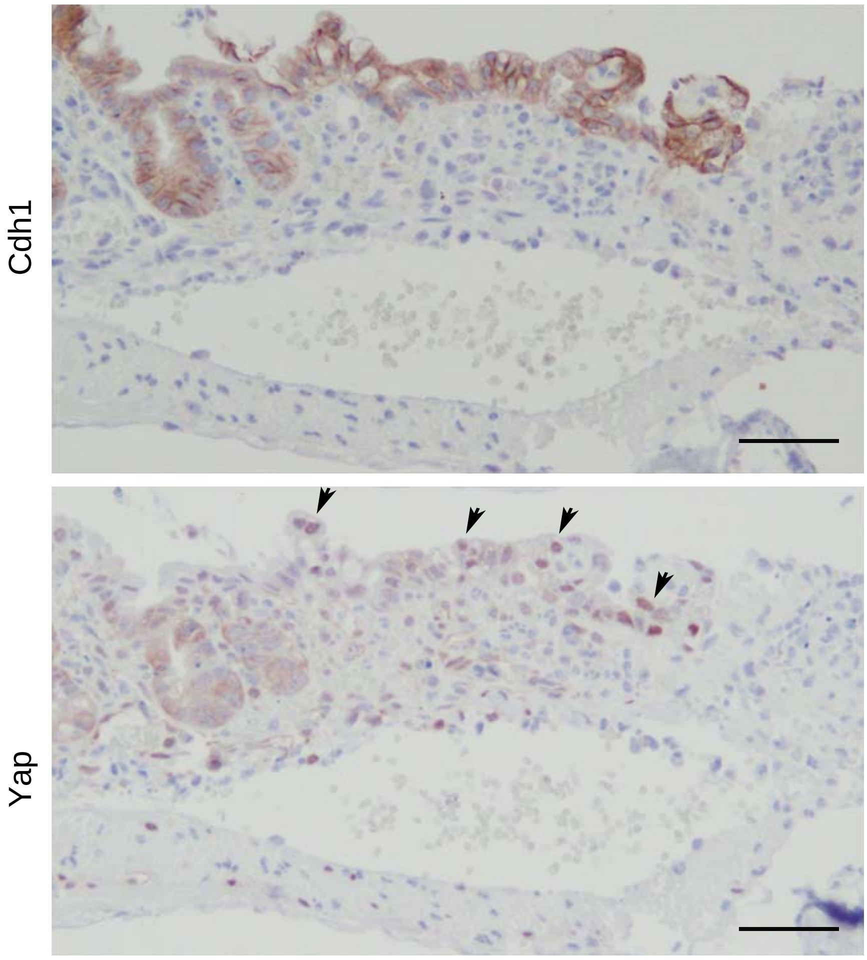


**Supplementary Figure S7**
**Immunohistochemical images of Yap and Cdh1 at 24 h after ischemia (corresponding to Fig. 3D).**
Arrows indicate cells with Yap nuclear translocation. Bar, 50 μm

**Supplementary Table S8** The periods after tamoxifen treatment in this study

| | weeks (w) after tamoxifen |
|---|---|
| Figure 2B | 4w |
| Figure 3 | 2w |
| Supplementary Figure S4 | 8w |
| Supplementary Figure S6A | 2w |
| Supplementary Figure S6B | 1w & 3w |

To express biosensors in the intestinal epithelial cells, we used Villin-CreERT2 mice, in which Cre is active in the intestinal epithelial cells, including stem cells. A previous lineage-tracing study using a knock-in mouse carrying a lox-STOP-lox-LacZ reporter showed that Villin-CreERT2 positive cells can give rise to all the cell types of intestinal epithelial cells, including stem cells. Therefore, once Villin-CreERT2 is activated by administration of tamoxifen, stem cells carrying a Cre-mediated recombined allele can give rise to all the cell types in the villi as early as 1 week after administration and keep producing cells carrying a recombined allele for 6 months after administration (Ref 16). Based on that report, administration of 2 mg tamoxifen/day for 3 days is sufficient to label a majority of ilial epithelial cells with biosensors, and stem cells expressing biosensors continue to produce epithelial cells with the same efficiency between 1 and 8 weeks after administration.
